# Microalgae empower skeletal muscle via increased force production and viability

**DOI:** 10.1126/sciadv.adw5786

**Published:** 2025-07-16

**Authors:** Xiang Wang, Claire Schirmer, Elena Totter, Simone Schuerle

**Affiliations:** Institute of Translational Medicine, Department of Health Sciences and Technology, ETH Zurich, 8092 Zurich, Switzerland.

## Abstract

Engineered skeletal muscle holds potential for tissue engineering and biohybrid robotics applications. However, current strategies face challenges in enhancing force generation while maintaining stability and scalability of the muscle, largely due to insufficient oxygenation and limited nutrient delivery. In this study, we present an engineering approach to address these limitations by coculturing *Chlamydomonas reinhardtii* (*C. reinhardtii*), a photosynthetic unicellular green microalga, with C2C12 myoblasts in a hydrogel matrix. Leveraging the photosynthetic activity of *C. reinhardtii*, our microalgae-empowered muscle (MAM) constructs exhibited superior contractility and almost three times higher active force generation compared to conventional muscle constructs. MAM showed higher cellular viability and reduced tissue damage, attributed to in situ oxygenation and nutrient supply provided by microalgal photosynthesis. In addition, improved myotube alignment was observed in MAM, which contributed to enhanced force generation. Our findings showcase the potential of photosynthetic microalgae as a functional component in engineered skeletal muscle, offering a solution to longstanding challenges in muscle engineering.

## Teaser

Symbiotic integration of microalgae enhances the performance of engineered skeletal muscle.

## INTRODUCTION

Skeletal muscle, comprising ~40% of human body mass (*1*), plays a fundamental role in movement (*2*), metabolism (*3*, *4*), and the maintenance of systemic homeostasis (*5*, *6*). The formation of skeletal muscle in vivo occurs naturally through the differentiation of myoblasts into multinucleated myotubes, under the regulation of myogenic transcription factors and external cues such as biochemical signals and mechanical stimuli (*7*). Inspired by this process, researchers have developed various biofabrication techniques to provide myoblasts with a microenvironment conducive to myogenesis, supplemented by appropriate stimuli, to engineer functional skeletal muscle constructs (*8*, *9*). These constructs exhibit structural characteristics resembling the bundled muscle fibers of native tissue and are capable of reproducing one of its most critical functions—externally controlled contraction. Consequently, these approaches have enabled the development of both biological and biohybrid skeletal muscle constructs, with diverse applications in tissue engineering (*10*, *11*), biohybrid robotics (*12*–*15*), cultured meat production (*16*, *17*), and drug testing models (*18*, *19*).

Despite the advancements, microscale engineered skeletal muscle constructs have yet to meet the demands of practical applications due to their limited size and force production. Consequently, the creation of large-scale, high-strength engineered muscle has long been a major goal in the field. However, scaling up engineered muscle introduces several challenges. One key issue is replicating the complex mechanical environment of native muscle tissue to achieve aligned myofiber arrangements, which are essential for forming anisotropic muscle fiber bundles with functional properties. Early efforts have used static stimulation to generate muscle constructs, which have been demonstrated to produce measurable force (*20*). To better mimic physiological conditions, researchers developed dynamic stimulation methods that further enhanced the contractility and force generation of skeletal muscle (*21*). Beyond mechanical stimulation approaches, optogenetic tools have been used to genetically modify myoblasts to express light-sensitive channelrhodopsins, which allow the control of muscle contraction through light stimulation while training the muscle in a minimally invasive manner (*22*). Nevertheless, these engineering strategies neglect the intrinsic limitations in engineered muscle constructs, which are insufficient oxygen and nutrient supply due to the absence of vascular and immune systems present in native muscle. Although some studies have explored the use of perfusion channels or implanted vascular networks to facilitate oxygen and nutrient transport within engineered constructs (*23*, *24*), these designs often require sophisticated bioreactor setups and continuous monitoring to generate precise flow dynamics (*25*), further complicating their application.

**Fig. 1.**
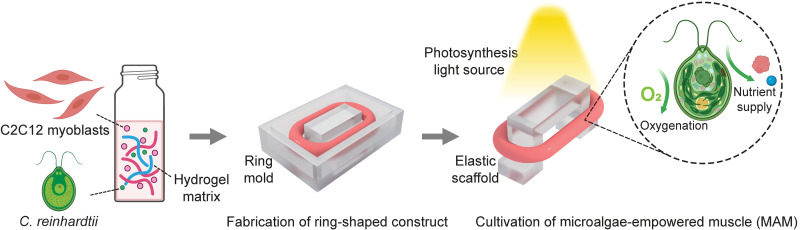
Schematic illustration of the fabrication process of MAM. C2C12 cells were mixed with *C. reinhardtii* in a hydrogel matrix. This mixture was then poured into the ring mold to create a ring-shaped muscle construct. The ring-shaped construct was transferred to an elastic scaffold to provide stretching stimuli to the construct. Under the exposure to light, the embedded microalgae released oxygen and provided nutrients to the muscle by means of photosynthesis.

**Fig. 2.**
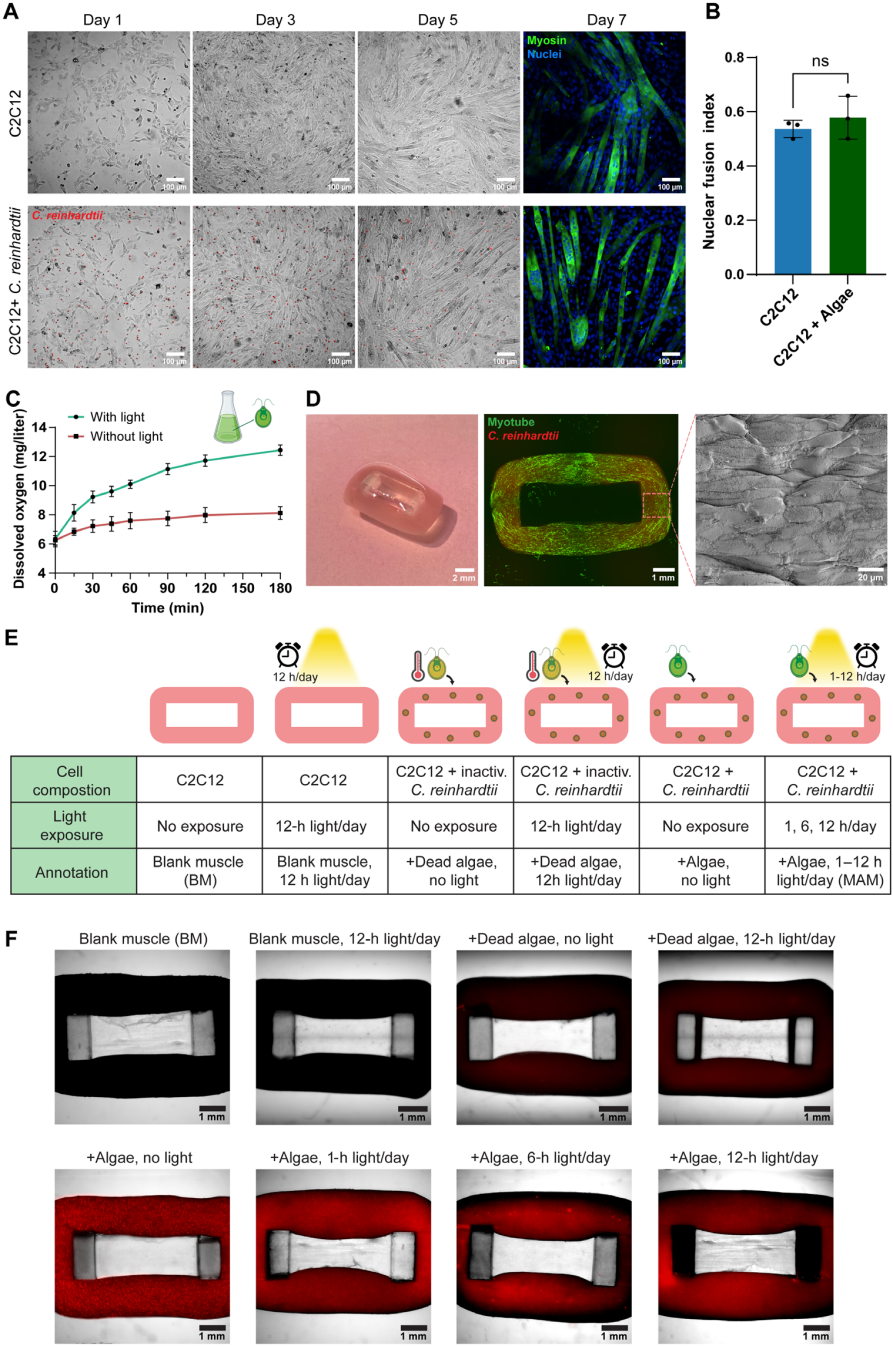
Cocultured *C. reinhardtii* do not hinder myogenic differentiation. (**A**) Fluorescence microscopy images of C2C12 cells or C2C12 cells + *C. reinhardtii* (cell number ratio 4:1) in 2D culture over a period of 7 days. On day 7, immunofluorescence staining of myosin in green using MF20 antibody and nuclei in blue using Hoechst was performed, and *C. reinhardtii* exhibited red autofluorescence of chlorophyll. (**B**) Quantification of the nuclear fusion index from 2D culture. Data are presented as means ± SD, *n* = 3 biological replicates. Statistical significance was determined using the nonparametric Mann-Whitney *U* test. Not significant (ns), *P* > 0.05. (**C**) Dissolved oxygen concentration in fresh microalgae culture over time with or without light exposure. Data are presented as means ± SD, *n* = 3 biological replicates. (**D**) Visualization of MAM at different scales: photograph, optical microscopy image (overlay of green fluorescence indicating myotubes and red fluorescence indicating microalgae), and SEM image of the surface of the MAM ring. (**E**) Summary of the different experimental conditions including cell composition, light exposure, and annotation of each group. (**F**) Optical microscopy images of representative samples from each group. All samples were imaged under both bright field and a fluorescence channel corresponding with the microalgal red autofluorescence, except for BM.

Here, we present a strategy to address the challenges of engineering skeletal muscle constructs by leveraging the photosynthetic microalga *Chlamydomonas reinhardtii* (*C. reinhardtii*). Unicellular green microalgae, such as *C. reinhardtii*, are well-known for their biocompatibility and sustainability (*26*, *27*). These unique properties have positioned microalgae as a promising tool in biomedical applications and biohybrid systems. For instance, biohybrid microrobots based on microalgae have been used to enhance the efficacy of photodynamic therapy by alleviating hypoxia in tumors (*28*) and regulating inflammatory bowel disease (*29*). In addition, studies have shown that microalgae embedded within a three-dimensional (3D) printed pattern released oxygen and enhanced the cellular viability of incorporated mammalian cell constructs (*30*). Notably, coculturing mammalian cells with microalgae was proven to be possible, which promoted the formation of thicker mammalian muscle tissues (*31*). Building on these insights, we developed microalgae-empowered muscle (MAM) by supplementing skeletal muscle constructs with *C. reinhardtii* and harnessing their photosynthetic activity (Figs. 1 and 2). Without relying on dynamic mechanical stimulation, MAM outperformed blank muscle (BM) lacking microalgae, demonstrating significantly enhanced contractility and force generation. In addition, MAM exhibited greater cellular viability and reduced tissue damage. These enhancements were attributed to the microalgae’s ability to optimize the cellular microenvironment by delivering in situ oxygenation and releasing essential nutrients. The optimized cellular microenvironment further improved the myotube alignment and altered differentiation dynamics of MAM, resulting in stronger and more viable skeletal muscle.

## RESULTS

### Myogenic differentiation is not hindered by cocultured *C. reinhardtii*

Although previous studies have demonstrated the applications of microalgae in boosting mammalian tissue culture, their impact on the functionality, especially the contractility and force generation of engineered muscle tissues, remains largely unexplored. To begin our study, we first investigated the feasibility of inducing myogenic differentiation of C2C12 myoblasts in a 2D coculture with *C. reinhardtii*. As shown in Fig. 2 (A and B), C2C12 cells, which were cocultured with *C. reinhardtii* at a cell number ratio of 4:1, exhibited normal myogenic differentiation over a period of 7 days, as indicated by the similar myotube structure (Fig. 2A) and a comparable nuclear fusion index (Fig. 2B) to those formed and quantified in monocultured C2C12 cells. These results indicate that the presence of microalgae does not interfere with myogenic differentiation through biochemical interactions. To explore the potential enhancement effects of microalgae in the context of muscle tissue, light exposure was incorporated to activate their photosynthetic activity. Given the photosynthetic oxygen-producing capability of microalgae, we first examined the effect of light exposure on microalgae alone. Light was provided at an intensity of 1800 lux, relatively lower than levels typically used for 3D algal cultures (*30*), to minimize potential phototoxic effects on C2C12 cells. At this light intensity, we measured the dissolved oxygen concentration in microalgae cultures over time to quantify the potential additional oxygen availability for cocultured cells (Fig. 2C). To fabricate microalgae-empowered skeletal muscle constructs, we adapted a ring-shaped muscle fabrication protocol (*20*) by embedding C2C12 cells and *C. reinhardtii* at the same ratio, 4:1, in a hydrogel matrix (Figs. 1 and 2D). Throughout the cultivation and differentiation period, the hydrogel matrix embedded with C2C12 and *C. reinhardtii* maintained its structural integrity, indicating that the hydrogel network remained intact and was not compromised by the high cell density (Fig. 2D). Meanwhile, a clear shrinkage of the ring-shaped construct was observed, and the muscle became increasingly attached to the elastic scaffold, suggesting an efficient myogenic differentiation of the myoblasts (fig. S1).

To identify the optimal conditions to fabricate MAM, we sought to examine a series of experimental parameters that are evaluated throughout the next sections (Fig. 2E). Because control experiments showed that 10 days of light exposure led to reduced C2C12 viability and impaired myogenic differentiation in 2D culture (fig. S2), we first assessed whether this phototoxic effect could affect muscle constructs by comparing BM cultured with or without light exposure. For constructs containing microalgae, we considered that embedding microalgae could alter matrix stiffness, influencing myogenic differentiation (*32*). To isolate this potentially confounding factor from the photosynthetic and metabolic activity of microalgae, we included two control groups embedded with heat-inactivated microalgae, achieved through incubation at 80°C for 1 hour (*33*). Their compromised viability was confirmed (fig. S3), and the constructs were cultured either in dark or under light exposure. To determine the effect of increasing illumination duration on active microalgae-embedded muscle constructs, we exposed them to 0 (no light), 1, 6, and 12 hours of light per day. All muscle constructs with active microalgae were cultured in differentiation medium (DM) supplemented with NH_4_Cl, a nitrogen source that supports microalgal metabolism (*34*) without negatively affecting C2C12 cells (*31*). As shown in Fig. 2F, microscopy images depict the obtained muscle constructs under different conditions. Constructs containing heat-inactivated microalgae exhibited negligible chlorophyll autofluorescence, while constructs with active microalgae displayed visible fluorescence in red. The fluorescence quantification of these images revealed a progressive decline in intensity with increasing daily illumination duration from 0 to 12 hours (fig. S4), likely due to the heat stress response of *C. reinhardtii* at 37°C under illumination. During the cultivation period of muscle constructs, the combined stress of light and elevated temperature led to reduced chlorophyll content and consequently diminished autofluorescence (*33*), suggesting the occurrence of photosynthesis.

### Skeletal muscle contractility and strength were enhanced by microalgal photosynthesis

**Fig. 3.**
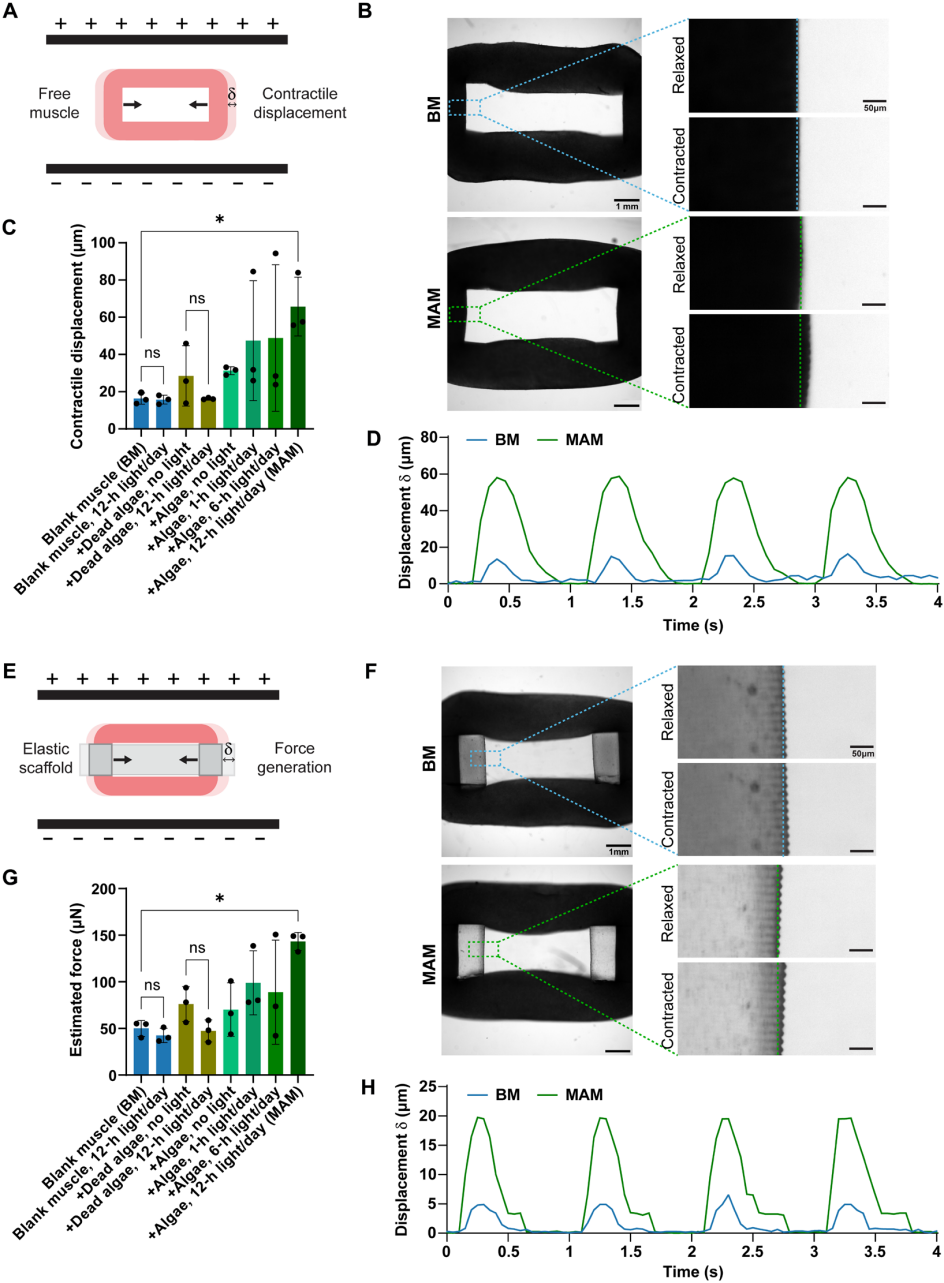
Skeletal muscle contractility and strength were enhanced by microalgal photosynthesis. (**A**) Schematic illustration of untethered muscle constructs exhibiting contraction under electrical stimulation. Muscle constructs were placed between two electrodes, which generated an electric field. (**B**) Quantification of the contractile displacement of the muscle constructs (without scaffold) based on the deformation measured in microscopy images. (**C**) Comparison of the maximum measured displacement of muscle rings cultured under different conditions. Data are presented as means ± SD, *n* = 3 biological replicates. Statistical significance was determined using the nonparametric Mann-Whitney *U* test. ns, *P* > 0.05 and *P ≤ 0.05. (**D**) Repeated contraction over time for electrical stimulation at 1 Hz of a single, representative sample for BM and MAM. (**E**) Schematic illustration of muscle constructs tethered to the elastic scaffolds exhibiting contraction, which results in the deformation of the scaffolds. (**F**) Displacement of the scaffold induced by muscle contraction measured in microscopy images. (**G**) Active force produced by muscle constructs, which was calculated on the basis of the maximum displacement (δ) of the scaffolds. Data are presented as means ± SD, *n* = 3 biological replicates. Statistical significance was determined using the nonparametric Mann-Whitney *U* test. ns, *P* > 0.05; *P ≤ 0.05. (**H**) Repeated scaffold displacement through muscle contraction over time for electrical stimulation at 1 Hz of a single, representative sample for BM and MAM.

To compare the contractility and force production of these muscle constructs, we used a customized setup to generate electric fields for stimulating muscle contraction (fig. S5). Muscle contraction was assessed in muscle constructs free from the scaffolds under electrical stimulation (Fig. 3A) where the displacement was measured over time (Fig. 3, B and D). The maximum contractile displacement of each muscle was then quantified for each of the samples (Fig. 3C). Similarly, the active contraction force was calculated on the basis of the deformation of scaffolds due to the contraction of tethered muscle (Fig. 3, E, F, and H), where the maximum displacement was used for the final force calculations (Fig. 3G). The results showed that BM constructs with and without light exposure exhibited comparable contraction and slightly reduced force generation under light, indicating that the phototoxic effects observed in 2D cultures were negligible in the 3D environment. A similar outcome was observed in constructs containing dead algae exposed to light. In addition, muscle constructs containing active or dead microalgae without light exposure exhibited slightly higher contractile displacement and active force generation compared to BM, possibly due to increased matrix stiffness caused by the presence of live/dead microalgae, which may promote muscle differentiation by activating mechanosensitive proteins (*32*). Constructs containing microalgae and exposed to 1 or 6 hours of daily light showed high variability among replicates despite notable improvements in contractility and force generation for one sample compared with BM and non-illuminated controls (Fig. 3, C and G). The best significance and reproducibility was obtained with 12 hours of daily light exposure. This construct generated ~143 μN of active force, which is nearly three times the 50 μN observed in BM. Based on these findings, microalgae-laden muscles exposed to 12 hours of light per day were designated as the optimized construct, referred to as the MAM.

**Fig. 4.**
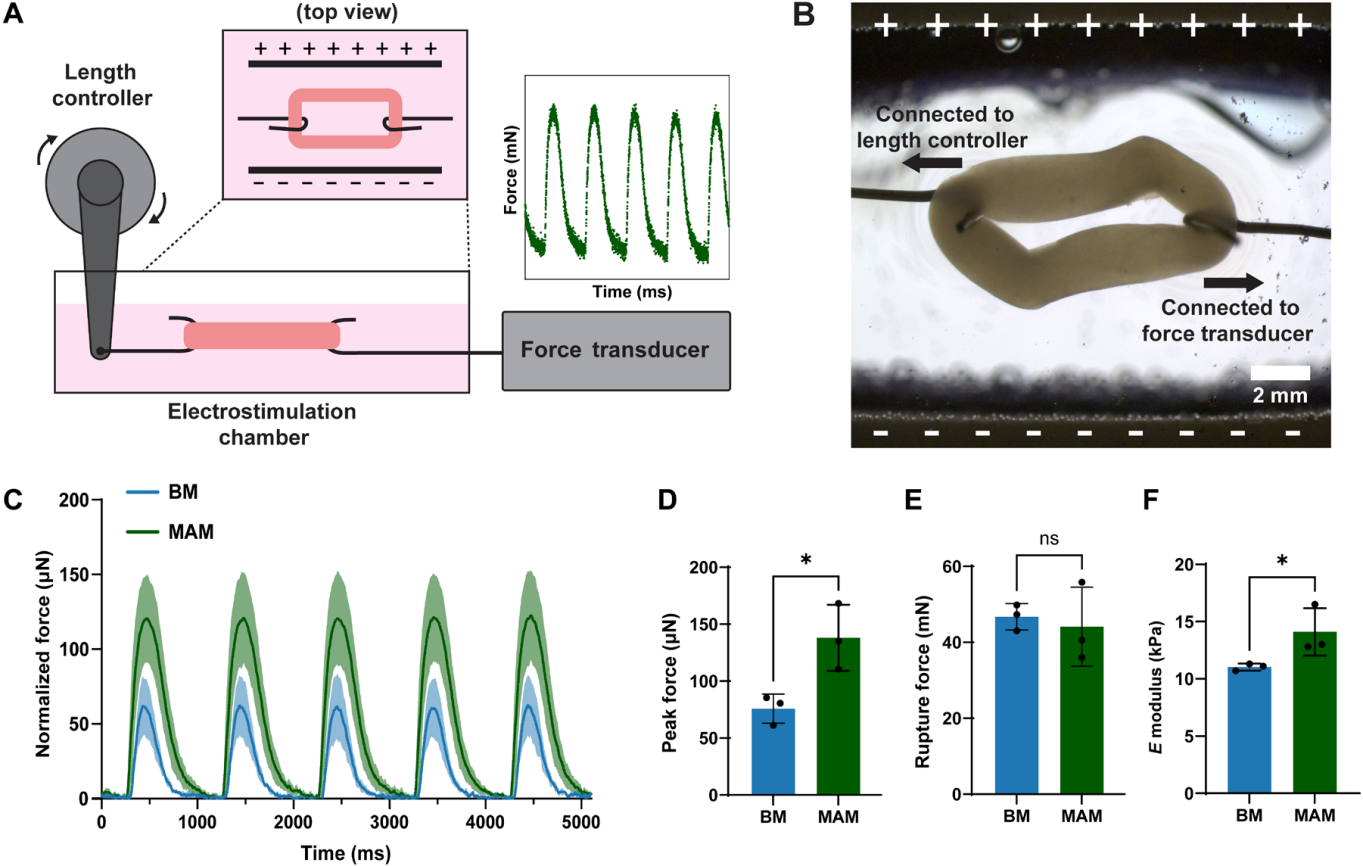
Force characterization of BM and MAM. (**A**) Schematic illustration of the force characterization setup. Muscle constructs were mounted between two wires, which were connected to a length controller and the force transducer, respectively. For active force measurements, the electrostimulation chamber was used to generate electric fields to stimulate the muscle constructs. The real-time active force generated by the muscle was measured by the force transducer. For passive tensile experiments, electric fields were turned off, and the length controller was used to stretch the muscle constructs to measure their mechanical properties. (**B**) Optical microscopy image of a muscle construct mounted between the length controller and force transducer wires. (**C**) Real-time active force over time generated by BM and MAM. Passive force was normalized to zero. (**D**) Peak active force generated by BM and MAM. (**E**) Rupture force of BM and MAM. (**F**) Elastic modulus (at a strain rate of 0.0263 s^−1^) of BM and MAM in the low-displacement regime. The data in (D) to (F) are presented as means ± SD, *n* = 3 biological replicates. Statistical significance was determined using the nonparametric Mann-Whitney *U* test. ns, *P* > 0.05; *P ≤ 0.05.

The mechanical properties of engineered muscle tissues are critical for their integration into biohybrid systems. To assess the mechanical performance of MAM, we used a real-time force characterization system, as shown in Fig. 4A and fig. S6. Muscle constructs were mounted between two flexible wires (Fig. 4B) and stimulated electrically to measure real-time active force generation (Fig. 4C). As shown in Fig. 4D, MAM produced significantly higher active force compared to BM. In addition, tensile testing using the same setup revealed that MAM exhibited similar rupture force (Fig. 4E) and significantly higher elastic modulus than BM (Fig. 4F), indicating enhanced mechanical strength and stiffness.

To investigate whether increasing microalgae content enhances muscle construct performance and to determine the optimal myoblast:algae ratio, we fabricated constructs with varying ratios (4:1, 3:1, 2:1, and 1:1). After exposing them to 12 hours of daily light, we assessed their contractile displacement, active force generation, and myotube thickness via immunofluorescence staining. As shown in fig. S8, contraction performance and myotube thickness of these constructs declined with increasing algae content, likely due to excessive microalgae interfering with myoblast differentiation. Because no significant difference was observed between 4:1 and 3:1 groups across all measurements, we selected the 4:1 ratio as optimal, as it supports robust muscle development while limiting the amount of microalgae, which are exogenous components that may hinder future biomedical translation of engineered muscle tissues.

### Microalgae support skeletal muscle by in situ oxygenation, release of nutrients, and reduction of cellular damage

**Fig. 5.**
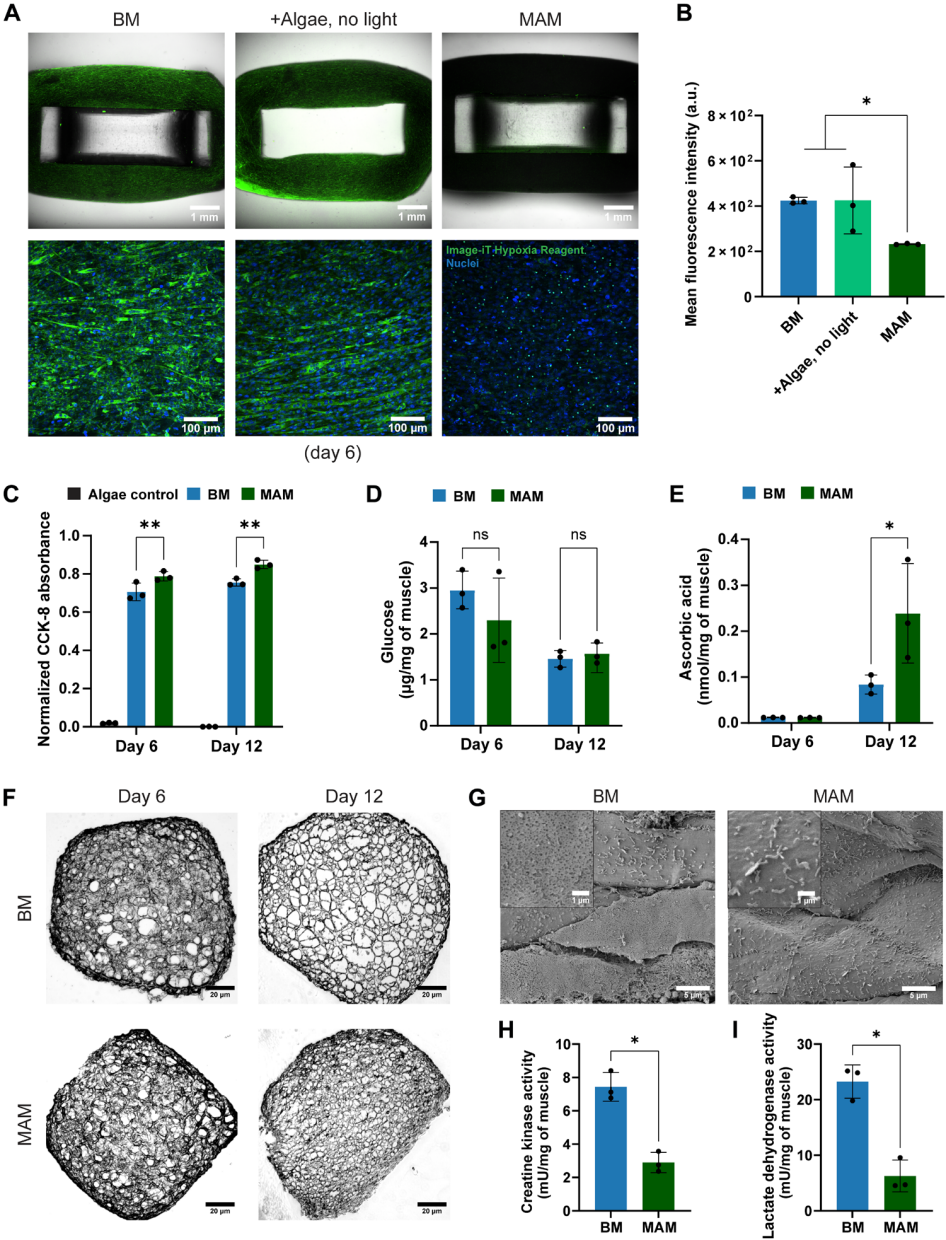
Microalgae support skeletal muscle by in situ oxygenation, release of nutrients, and reduction of cellular damage. (**A**) Fluorescence microscopy images of muscle constructs stained with Image-iT hypoxia reagent on day 6. Hypoxia reagent exhibited green fluorescence, whereas blue and red fluorescence represented nuclei and chlorophyll from microalgae, respectively. (**B**) Quantification of the mean fluorescence intensity of the hypoxia reagent. Data are presented as means ± SD, *n* = 3 biological replicates. Statistical significance was determined using the nonparametric Mann-Whitney *U* test, *P ≤ 0.05. (**C**) Analysis of cell viability of BM and MAM on day 6 and day 12 using CCK-8 assay. (**D**) Concentration of free glucose in BM and MAM on day 6 and day 12. (**E**) Concentration of ascorbic acid in BM and MAM on day 6 and day 12. Results are shown as the concentration of nutrients per milligram of wet samples. (**F**) Cross sections of BM and MAM on day 6 and day 12. The data in (C) to (E) are presented as means ± SD, *n* = 3 biological replicates. Statistical significance was determined using the two-way ANOVA test. ns, *P* > 0.05; *P ≤ 0.05; and **P < 0.01. (**G**) SEM images of the surface of MAM and BM and the zoomed-in views of the surface. SEM samples were prepared on day 12. (**H**) Activity of creatine kinase (CK) in BM and MAM on day 12. (**I**) Activity of lactate dehydrogenase (LDH) in BM and MAM on day 12. The data in (H) and (I) are presented as means ± SD, *n* = 3 biological replicates. Statistical significance was determined using the nonparametric Mann-Whitney *U* test, *P ≤ 0.05. a.u., arbitrary unit.

Having observed significantly increased contractility and force generation due to microalgal photosynthesis, we next sought to evaluate potential metabolic changes that microalgae photosynthesis may bring to the cellular microenvironment of muscle. Because oxygen production is one of the key advantages of microalgae (*35*), we assessed the oxygen levels within muscle constructs using a fluorescent dye, which precisely indicates hypoxia. As shown in Fig. 5 (A and B), fluorescence in MAM was significantly lower than BM on day 6, indicating MAM’s improved oxygenation due to the microalgae. In contrast, without light exposure, muscle containing microalgae displayed strong fluorescence of the hypoxia marker similar to those of BM. Muscle constructs stained with the hypoxia reagent were freshly cut to evaluate the spatial distribution of hypoxia via cross-sectional imaging. As shown in fig. S9, both BM and microalgae-embedded constructs without light (+Algae, no light) exhibited uneven hypoxia distribution, with stronger signals in the outer regions compared to the inner regions due to nonuniform distribution of myotubes (fig. S13). In contrast, MAM showed consistently low and homogeneous hypoxia levels across the entire cross section, suggesting improved oxygenation facilitated by microalgal photosynthesis. These results suggest that with the necessary conditions for photosynthesis, such as light and nitrogen source, microalgae are able to provide sufficient oxygen to their surrounding matrix.

Because oxygen levels can greatly influence the cellular viability of engineered muscle constructs (*36*), we evaluated the viability of MAM and BM constructs on day 6 and day 12 by using the Cell Counting Kit 8 (CCK-8). As shown in Fig. 5C, MAM exhibited significantly higher CCK-8 absorbance compared to BM at both days, reflecting overall greater cell viability. Notably, this increased viability was not contributed by the microalgae themselves, as the constructs embedded with only microalgae and cultured under the same condition showed negligible absorbance.

In addition to oxygen, nutrients are fundamental to maintaining a supportive microenvironment for muscle. Although the culture medium provides essential nutrients to engineered muscle, their availability is limited by passive diffusion, particularly for cells situated in deeper tissue regions. Microalgae are known to synthesize several critical nutrients via metabolic pathways, including glucose and ascorbic acid (*37*). Specifically, ascorbic acid has been reported to enhance myogenesis (*38*). To investigate the free nutrient levels within muscle constructs, we measured the concentrations of glucose and ascorbic acid in MAM and BM on day 6 and day 12. As shown in Fig. 5D, glucose concentrations in MAM and BM were comparable on both time points. Given that MAM contains more viable cells and microalgae, it should theoretically consume more glucose than BM. The comparable glucose levels suggest that the microalgae contributed to glucose release, thereby stabilizing intracellular glucose concentrations. In addition, ascorbic acid levels (Fig. 5E) were significantly higher in MAM than in BM on day 12, demonstrating the release of ascorbic acid from microalgae through metabolic degradation after day 6.

Adequate oxygen and nutrients improve the muscle microenvironment, reducing damage caused by hypoxia and limited nutrient supply. To further evaluate tissue integrity, we prepared cross sections of BM and MAM on day 6 and day 12 and quantified the porosity of their hydrogel matrix. As shown in Fig. 5F and fig. S10, the porosity of BM and MAM appeared similar on day 6. However, by day 12, two of the three MAM samples exhibited lower porosity compared to BM, although statistical analysis revealed no significant difference. In addition, after 12 days of cultivation, a large number of detached cells were observed in the culture plates of BM, whereas no visible detachment was found in MAM (fig. S11). SEM images revealed membrane perforations on the surface of BM, while MAM exhibited a smooth and intact surface morphology (Fig. 5G). To assess cellular damage, we analyzed the activity of creatine kinase (CK) and lactate dehydrogenase (LDH), two well-established tissue damage markers. Elevated CK and LDH activity is commonly associated with cellular membrane damage and metabolic stress, reflecting leakage of intracellular contents due to compromised viability (*39*). As shown in Fig. 5 (H and I), activities of CK and LDH in MAM were significantly lower than BM, indicating reduced cellular damage in MAM. Together, these results demonstrate that the presence of microalgae, under photosynthetic conditions, supports engineered skeletal muscle by in situ oxygenation, release of nutrients, and a decrease of cellular damage. Together, these results demonstrate that the presence of microalgae, under photosynthetic conditions, supports engineered skeletal muscle by in situ oxygenation, release of nutrients, and a decrease of cellular damage.

### MAM exhibited well-aligned myotube morphology and altered differentiation kinetics

**Fig. 6.**
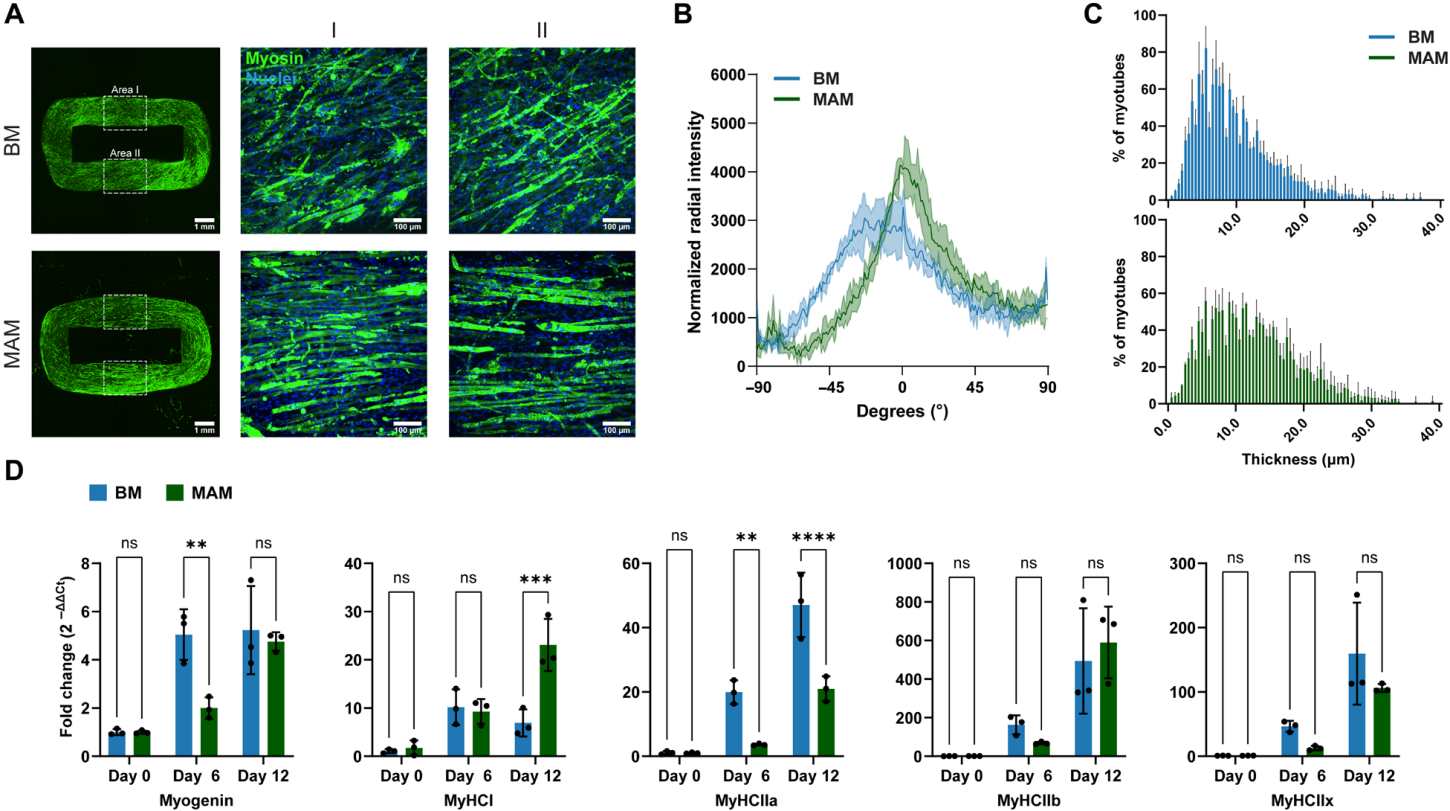
MAM exhibited well-aligned myotube morphology and altered differentiation kinetics. (**A**) Optical microscopy images of MAM and BM, with myosin stained in green using the MF20 monoclonal antibody which recognizes the heavy chain of myosin II, and nuclei stained in blue with Hoechst 33342. From the overview (left), Area I and Area II were selected as the region of interests for zoomed-in imaging (right). (**B**) Normalized radial intensity of the myotubes orientation using FFT. Data is presented as mean ± SD, *n* = 3 biological replicates. Statistical significance was determined using Kolmogorov-Smirnov analysis, *P ≤ 0.05. (**C**) Quantification of myotube thickness in MAM and BM. Data is presented as mean ± SD, *n* = 3 biological replicates. Statistical significance was determined using Kolmogorov-Smirnov analysis, not significant (ns) *P* > 0.05. (**D**) RT-qPCR analysis of myogenic biomarkers of MAM and BM normalized against the GAPDH housekeeping gene. Fold changes were calculated based on the corresponding Ct values of MAM and BM on day 0. Data is presented as mean ± SD, *n* = 3 biological replicates. Statistical significance was determined using two-way ANOVA. ns, *P* > 0.05; **P < 0.01; ***P < 0.001; and ****P < 0.0001.

The contractility and force production of skeletal muscle are normally influenced by various factors, including the dominant myofiber types and the alignment and thickness of myofibrils. To compare the myotube morphology of MAM and BM, we performed immunofluorescence staining to examine myosin, a key component of filaments in muscle fibers and a motor protein essential for skeletal muscle contraction (*40*). As shown in Fig. 6A, both MAM and BM exhibited strong fluorescence signals for myosin, indicating effective myogenic differentiation. However, MAM displayed well-aligned myotubes along the longitudinal contraction axis of the muscle ring, whereas myotubes in BM exhibited a lower degree of orientation in the direction of contraction with visible tilt or twist. This finding was further validated through angular analysis using fast Fourier transform (FFT) showing a high structural alignment of the MAM myotubes at 0° (longitudinal axis) (Fig. 6B) and a twisting of BM of up to 23°, which could directly be observed during muscle growth (fig. S12). In addition, the analysis of the average diameter of myotubes showed that MAM had a higher proportion of myotubes in the 20- to 30-μm range, while BM constructs were predominantly composed of thinner myotubes with diameters between 0 and 20 μm (Fig. 6C). Cryosections and spatial analysis of myotubes revealed a relatively homogeneous distribution of myotubes in MAM, whereas in BM, myotubes were predominantly localized closer to the outside/border of the ring section (fig. S13). These findings indicate that MAM is composed of more aligned, and slightly thicker, and homogeneously distributed myotubes, which could contribute to their enhanced contractility and force generation.

Skeletal muscles contain four major types of myofibers—type I, IIa, IIb, and IIx—each of them has different twitching speeds and contractile forces (*41*). Type I fibers are oxidative and fatigue-resistant and support sustained, aerobic activities, while type II fibers (IIa, IIb, and IIx) exhibit progressively faster twitching speeds and higher contractile forces, with IIb being the fastest (*42*). Typically, a slow-to-fast transition in fiber types, shifting from type I to type II, occurs during the maturation of muscle fibers under appropriate mechanical stimuli. Engineered skeletal muscle dominated by mature, fast-twitch myofibers is well-suited to meet the demands of applications such as biohybrid robotics and tissue engineering (*43*). To investigate the myofiber composition of MAM and BM, we performed reverse transcription–quantitative polymerase chain reaction (RT-qPCR) analysis at three time points during differentiation (days 0, 6, and 12). As shown in Fig. 6D, the myogenic regulatory factor myogenin, a key indicator of skeletal muscle differentiation (*44*), exhibited consistent up-regulation from day 0 to day 12 in both MAM and BM, ultimately reaching comparable levels on day 12. This suggests that myogenic differentiation was actively progressing in both MAM and BM. For type I myofibers (MyHCI), similar expression levels were observed in both MAM and BM on day 6, while significantly higher levels were detected in MAM by day 12. MyHCI represents a type of highly oxidative myofiber closely associated with aerobic muscle function (*45*). This result is consistent with what we observed in Fig. 5A, where the hypoxic marker was weaker in the MAM construct. Thus, the up-regulation of MyHCI in MAM may be attributed to enhanced oxygenation provided by the microalgae. In contrast, the three type II myofibers showed noticeably lower expression in MAM compared to BM on day 6, suggesting a delayed slow-to-fast transition in MAM. By day 12, however, with the exception of MyCHIIa, both MyHCIIb and IIx myofibers in MAM had almost completely caught up with the expression levels observed in BM. MyHCIIb, as the fastest contracting myofiber, contributed substantially to the improved contractility and force production of MAM. Furthermore, the up-regulation of myogenic markers in MAM predominantly occurred between day 6 and day 12, demonstrating delayed but accelerated differentiation in the later stages. This delay could be attributed to possible competition between microalgae and C2C12 myoblasts in the early stages of differentiation (before 6 days). By providing oxygen and releasing nutrients such as ascorbic acid into the myotubes environment, microalgae ultimately stimulated differentiation. To ensure the accuracy of the results, we used an additional housekeeping gene as a control, which showed a similar trend (fig. S14).

## DISCUSSION

This study highlights the potential of microalgae to enhance the functionality and viability of engineered skeletal muscle by addressing key challenges in the muscle, including poor oxygenation, limited nutrient supply, and tissue damage. By incorporating microalgae *C. reinhardtii* into the fabrication process of engineered skeletal muscle and supporting it with light and nitrogen for photosynthesis (Fig. 1), we fabricated MAM. Our results demonstrate that the cocultured microalgae do not hinder myogenic differentiation of the embedded myoblasts (Fig. 2). Without the use of dynamic mechanical stimulation or bioreactor-driven perfusion systems, MAM demonstrated nearly three times the contractility and force generation compared to BM without microalgae (Fig. 3) and better mechanical properties (Fig. 4). Our investigation revealed that microalgae supported muscle function by releasing oxygen and essential nutrients through photosynthesis. This process enhanced overall viability and minimized tissue damage in MAM (Fig. 5). Moreover, the formation of thicker myotubes and improved alignment were key factors contributing to the higher force generation observed in MAM. Because both MAM and BM were subjected to the same static stretching from the elastic scaffolds, the well-aligned myotubes observed in MAM were unlikely to result from mechanical cues. Chemokine receptors expressed in myoblasts, known to drive chemotactic migration during the myogenesis process (*46*), may play a role in this phenomenon. The cellular microenvironment in MAM, optimized by microalgae, provided a more uniform distribution of oxygen and nutrients compared to BM. This favorable environment may reduce the tendency of myoblasts to migrate toward the muscle construct’s edges, where concentrations of oxygen and nutrients that diffused from the medium are typically higher. Although MAM showed delayed myogenic differentiation during the early stages, this delay diminished over time due to the optimized cellular microenvironment, which resulted in faster late-stage differentiation, producing more contractile skeletal muscle (Fig. 6).

Our study demonstrates that, beyond external interventions such as stimulating skeletal muscle mechanically or artificially perfusing nutrients, incorporating microalgae into the muscle can be a promising strategy to enhance the contractility and viability of engineered muscle. As a photosynthetic microorganism classified as “generally recognized as safe,” *C. reinhardtii* pose minimal risks for humans, making this approach highly promising for health care or other human-related applications. Nonetheless, there is substantial room for further exploration. Future studies could investigate the long-term stability of microalgae-empowered muscle constructs or extend this strategy to other cell types, such as cardiomyocytes. In addition, this study not only provides a standalone solution for engineering functional muscle constructs but also opens avenues for integration with other advanced biofabrication strategies. In future work, microalgae-empowered muscle could be combined with techniques such as perfusion channels or vascular networks to further enhance nutrient delivery and enable scalability toward thicker and larger constructs.

## MATERIALS AND METHODS

### Cell culture

C2C12 mouse muscle myoblasts (Sigma-Aldrich, 91031101) were cultured in growth media (GM) consisting of Dulbecco’s modified Eagle’s medium (DMEM) with high glucose and pyruvate (Thermo Fisher Scientific, 11995065) supplemented with 10% (v/v) fetal bovine serum (Biowest, S1810), 1% (v/v) l-glutamine (Thermo Fisher Scientific, 25030081), and 1% (v/v) penicillin/streptomycin (Thermo Fisher Scientific, 15070063). The cells were maintained in a humidified incubator at 37°C with 5% CO_2_, passaged upon reaching 70 to 80% confluency, and used for experiments up to passage 10 to ensure cell quality and consistency. The green microalgae *C. reinhardtii* (CC-125 wild-type mt + [137c], Chlamydomonas Resource Center, University of Minnesota) were provided by the Sustainable Food Processing Laboratory of ETH Zurich and cultured in tris-acetate-phosphate (TAP) medium (Thermo Fisher Scientific, A1379801) at 25°C under 12-hour light/12-hour dark cycles. Light was provided at an intensity of 1800 lux using a light-emitting diode (LED) panel comprising 225 diodes, including 60 red LEDs (λ = 730 nm), 5 far-red LEDs (λ = 850 nm), and the remainder emitting white light.

### Fabrication of MAM and BM

The fabrication method of MAM was adapted from an established protocol (fig. S1) (*20*). Briefly, a minimum of 3 × 10^6^ C2C12 cells and 7.5 × 10^5^ *C. reinhardtii* cells were harvested, mixed, and resuspended in 60 μl of cold GM supplemented with 6-aminocaproic acid (1 mg/ml; Sigma-Aldrich, A2504) (GM^+^). To this cell suspension, 6 μl of thrombin stock solution [Sigma-Aldrich, T4648; Milli-Q water (100 U/ml) with 0.1% (w/v) bovine serum albumin (BSA), 90 μl of Matrigel (Corning, 354248), and 156 μl of fibrinogen solution (Sigma-Aldrich, F8630; 8 mg/ml in GM^+^)] was sequentially added and mixed thoroughly. For each construct, 200 μl of the mixture was immediately added to a rectangular ring-shaped polydimethylsiloxane (PDMS) mold (fig. S15A) placed in a six-well plate and incubated at 37°C for 1 hour to allow for gelation. Subsequently, 5 ml of prewarmed GM^+^ containing 50 μM NH_4_Cl was added to each well. After incubating for 48 hours, each muscle ring was carefully loaded onto a PDMS elastic scaffold (fig. S15B) using a sterilized tweezer. Medium was replaced by 4 ml of differentiation medium (DM) containing DMEM supplemented with 10% (v/v) horse serum (Thermo Fisher Scientific, 26050070), 1% (v/v) l-glutamine, 1% (v/v) penicillin/streptomycin, 6-aminocaproic acid (1 mg/ml), insulin-like growth factor 1 (IGF-1) (5 ng/ml; human IGF-I recombinant protein, PeproTech), and 50 μM NH_4_Cl. Muscle rings were maintained at 37°C under 12-hour light/12-hour dark cycles for at least 10 days. DM was changed every day before applying light exposure. The fabrication process of BM followed the same protocol, without the use of *C. reinhardtii*, NH_4_Cl, and light exposure.

### Electrical stimulation and force calculation

The electrical stimulation setup for muscle consisted of a function generator (Keysight EDU33212A), a signal amplifier (Crown D-150A Series II), a 100-μF capacitor to minimize electrolysis, an oscilloscope (Keysight DSOX2004A), and a homemade electrostimulation chamber equipped with two parallel graphite rods (Thermo Fisher Scientific, 040765.KM) serving as electrodes. Muscle constructs were positioned in the chamber between the electrodes. The circuit was calibrated to generate a field intensity of 21.6 V/cm with a frequency of 1 Hz and a pulse width of 50 ms, resembling the waveform shown in fig. S5. Videos were recorded using an inverted spinning disk confocal microscope (Nikon Eclipse Ti2, equipped with a Yokogawa CSU-W1 unit and a Hamamatsu C13440-20CU digital complementary metal-oxide semiconductor camera).

To estimate the force generation of muscle constructs under stimulation, muscle constructs confined to elastic scaffolds were subjected to electrical stimulation. Snapshots from contraction videos were analyzed using Fiji software to measure the maximum displacement of the muscle. Force generation (*F*) was calculated on the basis of the Euler-Bernoulli beam theory, as follows

$$F=\frac{8EI\delta_{\text{max}}}{lL^{2}}$$

In this equation, *E* represents the Young’s modulus of the elastic scaffold (PDMS, with a monomer-to-crosslinker ratio of 20:1), reported to be 255 kPa (*21*), *I* is the moment of inertia of the bending beam, δ_max_ is the maximum displacement of the scaffold, *l* is the distance from the middle of muscle loading point to the top of the scaffold (measured using the microscope), and *L* is the length of the scaffold.

### Real-time force characterization

Real-time force characterization of the muscle constructs was performed using the Aurora Force Transducer System (fig. S6). BM and MAM were carefully removed from the elastic scaffolds and mounted between two flexible wires with tweezers. The wires were connected to a length controller and a force transducer, respectively. For active force measurement, the constructs were placed in an electrostimulation chamber integrated with the setup, where electrical pulses were applied to induce muscle contraction. For tensile testing, electric fields were turned off, and the length controller was programmed to stretch the muscle over a defined distance, allowing measurement of passive force and calculation of mechanical properties.

For the peak force, the mean of five sequential local maxima was taken, and their baseline was subtracted for each sample. The rupture force was determined by taking the maximum values of the overall curves. The elastic modulus was calculated by dividing the nominal stress with the nominal strain of each curve at a known initial displacement, with a displacement rate of 0.25 mm/s and an initial elongation (*L*_0_) of 9.5 mm.

### Evaluation of oxygenation and free nutrients in muscle

Oxygenation within the muscle constructs was assessed using the Image-iT Green Hypoxia Reagent (Thermo Fisher Scientific, I14833) following the manufacturer’s protocol. Dissolved oxygen concentrations were measured with the Mini Lab Grade Dissolved Oxygen Probe (Atlas Scientific). Nutrient levels were evaluated using ascorbic acid assay kit (Sigma-Aldrich, MAK505) and glucose assay kit (Sigma Aldrich, GAGO20) following the manufacturer’s protocol. All results were normalized to the mass of the samples.

### Viability assay

The relative viability of MAM and BM on days 6 and 12 was assessed using the CCK-8 (Dojindo, CK04) following the manufacturer’s protocol. Briefly, MAM, BM, and ring-shaped constructs containing only microalgae (algae control) were incubated in 500 μl of DMEM basic medium containing 10% (v/v) CCK-8 reagent at 37°C for 1 hour. After incubation, 100 μl of the supernatant was transferred to a 96-well plate in technical triplicates, and the absorbance at 450 nm was measured using a microplate reader (Tecan Spark Reader). All results were normalized against the absorbance of DMEM medium.

### Activity assays of tissue damage–related enzymes

Tissue damage–related enzymes were evaluated using the Lactate Dehydrogenase Activity Assay Kit (Sigma-Aldrich, MAK066) and Creatine Kinase Activity Assay Kit (Sigma-Aldrich, MAK116) according to the manufacturer’s protocol. Before analysis, BM and MAM were mechanically homogenized while frozen in liquid nitrogen, resuspended in the appropriate assay buffer, and centrifuged. The resulting supernatant was collected and used for the assays.

### Immunofluorescence staining

MAM and BM were washed three times with phosphate-buffered saline (PBS) for 5 min each, fixed in 4% paraformaldehyde (in PBS) for 30 min at room temperature, and washed three times in PBS. Fixed samples were permeabilized in 0.2% (v/v) Triton X-100 in PBS for 15 min on a shaker at 4°C, followed by three washes in PBS. Samples were blocked for 30 min at 4°C in 3% (w/v) bovine serum albumin (BSA) in PBS and then incubated with myosin 4 monoclonal antibody MF-20 (2.5 ng/ml in PBS–3% BSA) conjugated with Alexa Fluor 488 (Thermo Fisher Scientific, 53-6503-82) overnight at 4°C. After three washes in PBS, nuclear staining was performed using Hoechst 33342 (2 μg/ml; Thermo Fisher Scientific, H3570) for 10 min at room temperature, with three subsequent washes in PBS before imaging.

### Cryosectioning

After immunofluorescence staining, muscle constructs were embedded in optimal cutting temperature (OCT) compound (Tissue-Tek) and frozen in isopentane cooled by liquid nitrogen. Cross sections (10 μm) were prepared using a cryostat (Cryostar NX70, Thermo Fisher Scientific).

### Reverse transcription–quantitative PCR

To perform RT-qPCR, MAM and BM were collected and snap-frozen on day 0 (after gelation), day 6, and day 12. To extract RNA, samples were mechanically homogenized while frozen in liquid nitrogen and subsequently homogenized again in TRIzol Reagent (Thermo Fisher Scientific, 15596026). Total RNA was extracted using the RNeasy Mini Kit (QIAGEN, 74104) according to the manufacturer’s protocol. For each sample, 500 ng of RNA was reverse-transcribed into cDNA using the RevertAid First Strand cDNA Synthesis Kit (Thermo Fisher Scientific, K1621). The qPCR was conducted using PowerUp SYBR Green Master Mix (Thermo Fisher Scientific, A25742), and the QuantStudio 1 Real-Time PCR System (Applied Biosystems). Glyceraldehyde-3-phosphate dehydrogenase (GAPDH) and Ribosomal Protein S12 (RPS12) were used as housekeeping genes for normalization. All primers were obtained from Microsynth AG according to reported nucleotide sequences (*21*, *47*) and are listed in table S1. All reactions were performed in triplicate to ensure reproducibility. Relative gene expression levels were calculated using the ΔΔ*Ct* method. Fold changes were calculated using MAM and BM on day 0 as their corresponding controls.

### Image analysis

All image analyses were performed in Fiji (ImageJ 1.54f) using the integrated functions and methods. The Fusion Index was analyzed using Z-stack images with maximum intensity projections of either C2C12 cells alone or C2C12 + *C. reinhardtii* cocultured cells both after 7 days of differentiation. The images were binarized, and masks were generated using the myosin images. Subsequently, the number of nuclei was counted first inside the myosin mask and then in total.

The fluorescence of the hypoxic reagent was determined by measuring the mean intensity of maximum intensity z-projection images. The mean of three images was taken for each sample. Biological triplicates for each group (BM and MAM) were analyzed.

The alignment of myotubes was analyzed using FFT adapted from an established protocol (*20*). Z-stack images of each sample were imported, and maximum intensity projections were generated. FFT was applied to the images and circular regions of interest (ROIs) were selected around the center, excluding high frequency noise. To quantify the myotube alignment distribution, the Oval Profile plugin was applied to the selected ROIs using the radial sum setting to extract alignment data along 180° (at 1° increments). For each sample, two images were analyzed, and their average was treated as a single data point. Biological triplicates for each group (BM and MAM) were analyzed.

Myotube thickness was analyzed using Z-stack images of each sample with maximum intensity projections. Contrast was enhanced with 0.35% saturated pixels and histogram equalization. Local thresholding was applied using the Phansalkar method (radius 15). Noise was reduced by removing outliers (diameter < 5 μm) for bright and dark regions. Myotube thickness was calculated using the integrated masked local thickness function, and histograms were generated for further analysis.

### Statistical analysis

All data were expressed as means ± SD. Statistical analysis was conducted using GraphPad Prism 10 software. For comparisons between two groups, the nonparametric Mann-Whitney *U* test was used. Two-way analysis of variance (ANOVA) followed by Sidak’s post hoc test was used to analyze the interaction between two independent variables with more than two groups. Kolmogorov-Smirnov test was used to compare the distributions of histograms or line plots. Significance levels were indicated as not significant (ns), *P* > 0.05; *P ≤ 0.05; **P < 0.01; ***P < 0.001; and ****P < 0.0001. All experiments were performed in biological replicates (*n* = 3).
