# Supplementary material for: Microalgae empower skeletal muscle via increased force production and viability
Source: Sci Adv. 2025 Jul 16;11(29):eadw5786. doi: 10.1126/sciadv.adw5786 (PMC12266117; doi:10.1126/sciadv.adw5786)
Supplement: Supplementary file 1 — Supplementary Materials and Methods Figs. S1 to S15 Table S1 Legend for movie S1 [file sciadv.adw5786_sm.pdf]

Science Advances
AAAS

Supplementary Materials for

Microalgae empower skeletal muscle via increased force production and viability

Xiang Wang *et al.*

Corresponding author: Simone Schuerle, simone.schuerle@hest.ethz.ch

Sci. Adv. **11**, eadw5786 (2025)
DOI: 10.1126/sciadv.adw5786

The PDF file includes:

Supplementary Materials and Methods
Figs. S1 to S15
Table S1
Legend for movie S1

Other Supplementary Material for this manuscript includes the following:

Movie S1

Supplementary Materials and Methods

Fabrication of ring molds and elastic scaffolds

The ring molds and elastic scaffolds were fabricated through a mold printing and casting process (Fig. S2). Positive pre-molds for the ring molds and negative pre-molds for the scaffolds were designed and printed using a digital light processing (DLP) 3D printer. A rubber compound (Mold Max 30 Silicones, Smooth-On) was used to cast the rubber molds. Subsequently, epoxy resin (EpoxAcast 690, Smooth-On) was used to fabricate epoxy molds. These epoxy molds were treated with air plasma and surface-coated with chlorotrimethylsilane for silanization to enhance hydrophobicity. Sylgard 184 silicone elastomer (Dow) was used to fabricate the final ring molds and elastic scaffolds by mixing the monomer and crosslinker at ratios of 10:1 for the molds and 20:1 for the scaffolds. The resulting mixtures were cured overnight in an 80°C oven. Before muscle construct fabrication, ring molds and scaffolds were treated with air plasma to enhance hydrophilicity and sterilized by autoclaving.

Scanning electron microscopy (SEM) imaging

MAM and BM on day 12 were fixed in 2.5% glutaraldehyde overnight. The next day, the stiffened samples were sectioned into pieces at different orientations using a fresh scalpel, including cross-sections (perpendicular cuts) and longitudinal cross-sections (tangential cuts). The sections were osmicated with 1% OsO_4, dehydrated through an ascending ethanol series, and dried using critical point drying with liquid CO_2 (Tousimis CPD 931). The dried samples were mounted onto SEM stubs and sputter-coated with a 5 nm layer of Pt/Pd using planetary rotation mode for uniform coating. Imaging was performed using a Thermo Fisher Scientific Magellan 400i field emission SEM, with secondary electron detection at an accelerating voltage of 2 kV.

Spatial analysis of hypoxia in muscle constructs

After staining the muscle constructs with hypoxia reagent and cutting them into sections, microscopy images were taken as Z-stacks of the bright field and the fluorescence channel. The bright field images were used to determine the outer border of the ring sections (referred to as "*outer*" region). Subsequently, a circular center region was defined corresponding to approximately 1/13 of the section's diameter (averaged over all analyzed sections). This size was chosen to have a small area which would nevertheless be large enough to account for spatial variations in hypoxia intensity. The area between the center circle and the outer border was interpolated to create a total of four regions: "*center*", "*inner*", "*middle*" and "*outer*". The mean fluorescence intensity was measured for all four regions, and the *inner*, *middle* and *outer* regions were normalized to the *center* intensity value to represent the ratio of intensity increase or decrease between the different regions.

Porosity study of BM and MAM on day 6 and day 12

BM and MAM muscle rings were cryosectioned (10 µm) on day 6 and day 12. These cross sections were imaged as bright field Z-stacks. After minimum-intensity projection of the stacks, the images were binarized and the total area of the cross section as well as the sum of all pore areas were measured. The pore area was divided by the total cross section area to get the porosity (%) of the individual section.

Spatial analysis of myotube distribution

The spatial analysis of myotube distribution was performed as follows: Microscopy images of cryosections were taken as Z-stacks of the bright field and the fluorescence channel. The bright field images were used to determine the outer border of the ring sections (referred to as "*outer*" region). Subsequently, a circular center region was defined corresponding to approximately 1/7 of the section's diameter (averaged over all analyzed sections). This size was chosen to have a large enough area which would account for spatial variations in intensity stemming from individual, stained myotubes. The area between the center circle and the outer border was interpolated to create a total of four regions: "*center*", "*inner*", "*middle*" and "*outer*". The mean fluorescence intensity was measured for all four regions, and the *inner*, *middle* and *outer* regions were normalized to the *center* intensity value to represent the ratio of intensity increase or decrease between the different regions.

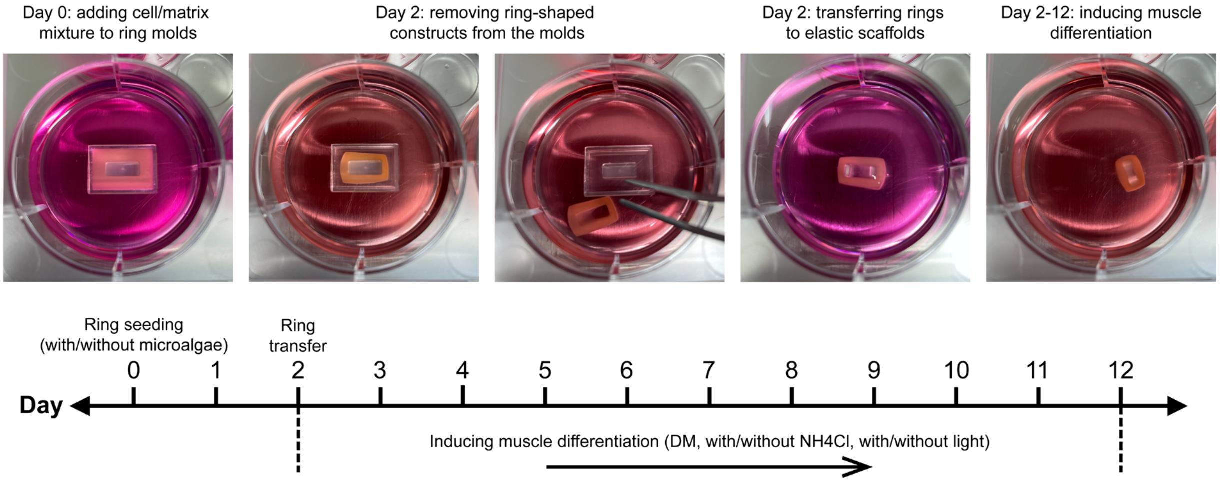

Figure S1. Photographs of Muscle Ring Fabrication and Differentiation process with the corresponding Timeline.

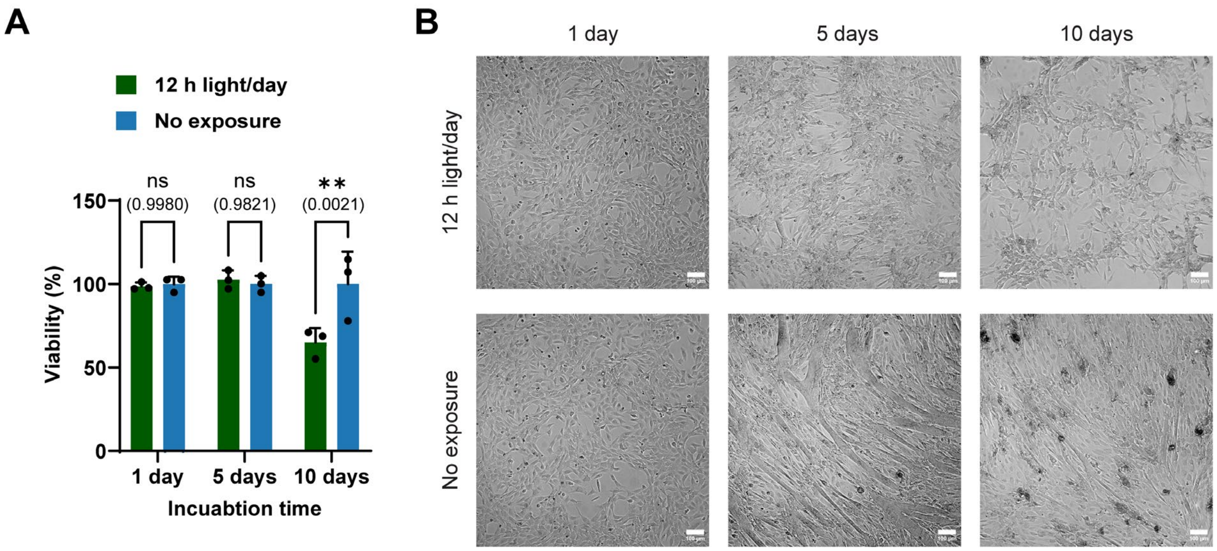

Figure S2. Evaluation of C2C12 viability and differentiation under light exposure. (A) Measurement of C2C12 cell viability after 1, 5, and 10 days of light exposure (12 hours per day). Cell viability was calculated using the CCK-8 assay and normalized to non-illuminated cells at each corresponding time point, which was considered as 100% viable. Data is presented as mean ± SD, N = 3 biological replicates. Statistical significance was determined using the two-way ANOVA test. Not significant (ns) $P > 0.05$, $^{**}P \leq 0.01$. **(B)** Optical microscopy images of C2C12 cells cultured in differentiation medium (DM) and incubated for 1, 5, and 10 days, with or without light exposure. C2C12 cells cultured without light exposure differentiated into myotubes over time, whereas light-exposed cells showed inhibited differentiation.

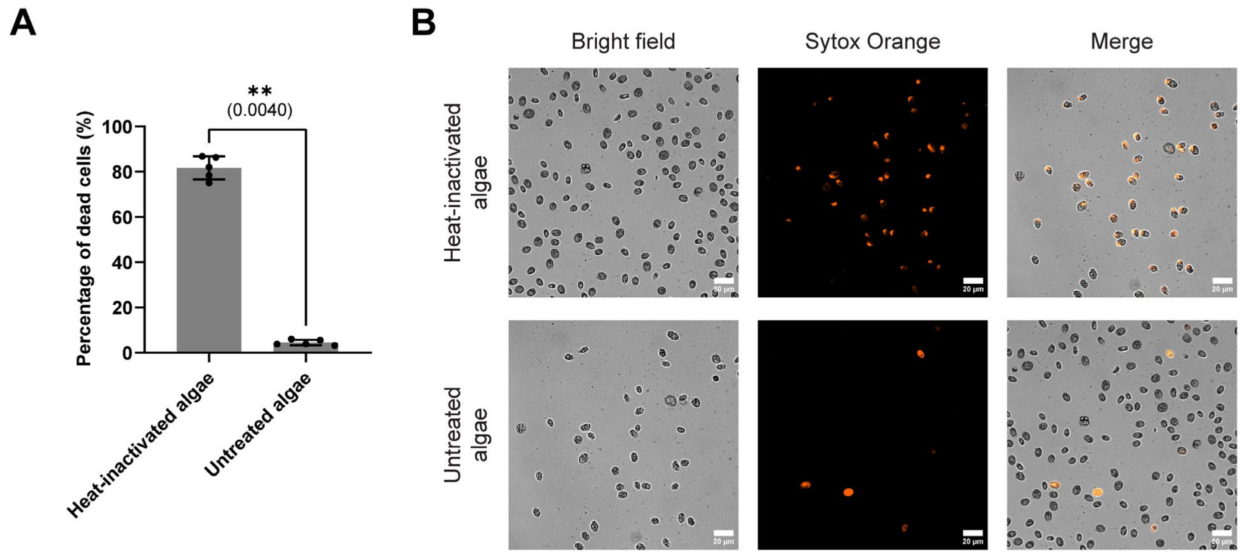

Figure S3. Viability test of microalgae. (A) Quantification of dead cell percentage (%) in heat-inactivated and untreated microalgae, confirmed by Sytox Orange dead cell staining. Data is presented as mean ± SD, N = 5 biological replicates. Statistical significance was determined using the nonparametric Mann–Whitney U test, **P < 0.01. **(B)** Optical microscopy images of heat-inactivated and untreated microalgae stained with Sytox Orange.

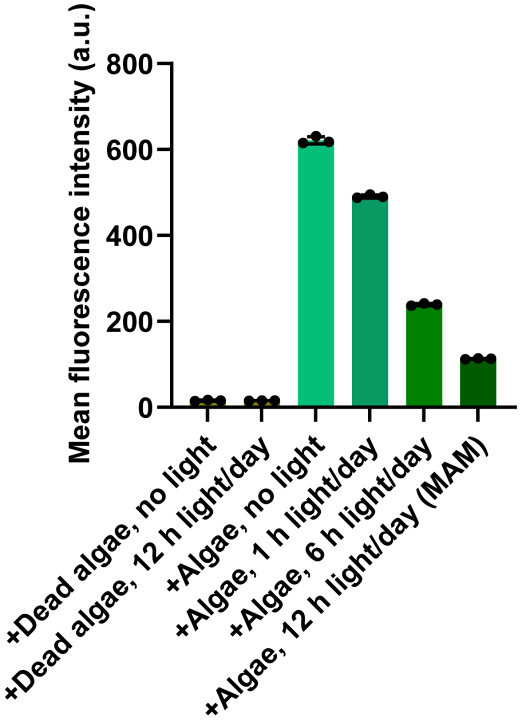

Figure S4. Quantification of Chlorophyll fluorescence intensity of muscle constructs under different conditions. The samples were imaged on day 12.

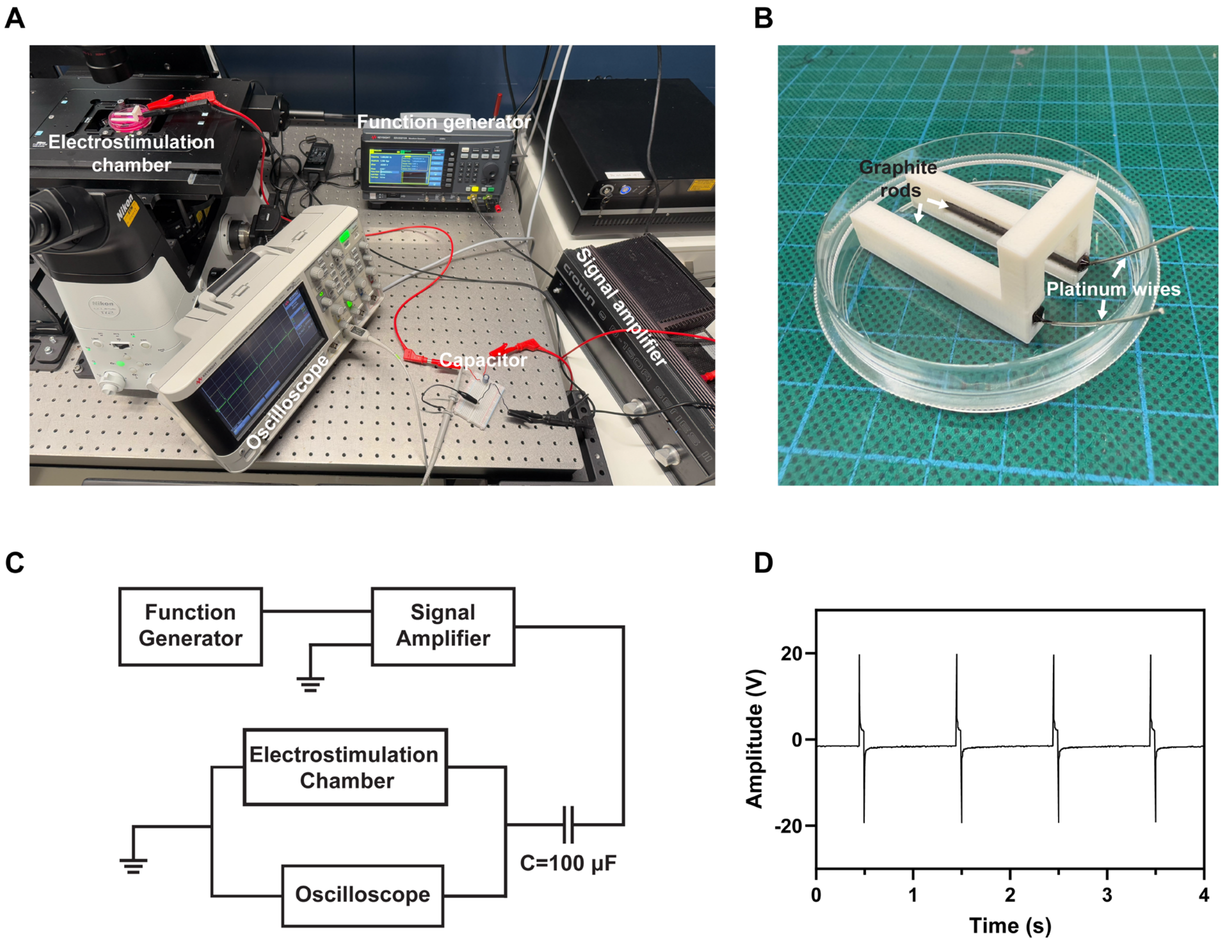

Figure S5. Electrical stimulation setup. (A) Photograph of the complete electrical stimulation setup. **(B)** Photograph of the electrostimulation chamber. **(C)** A schematic diagram of the circuit used in the electrical stimulation setup. **(D)** Output signal waveform for electrical stimulation at a frequency of 1 Hz.

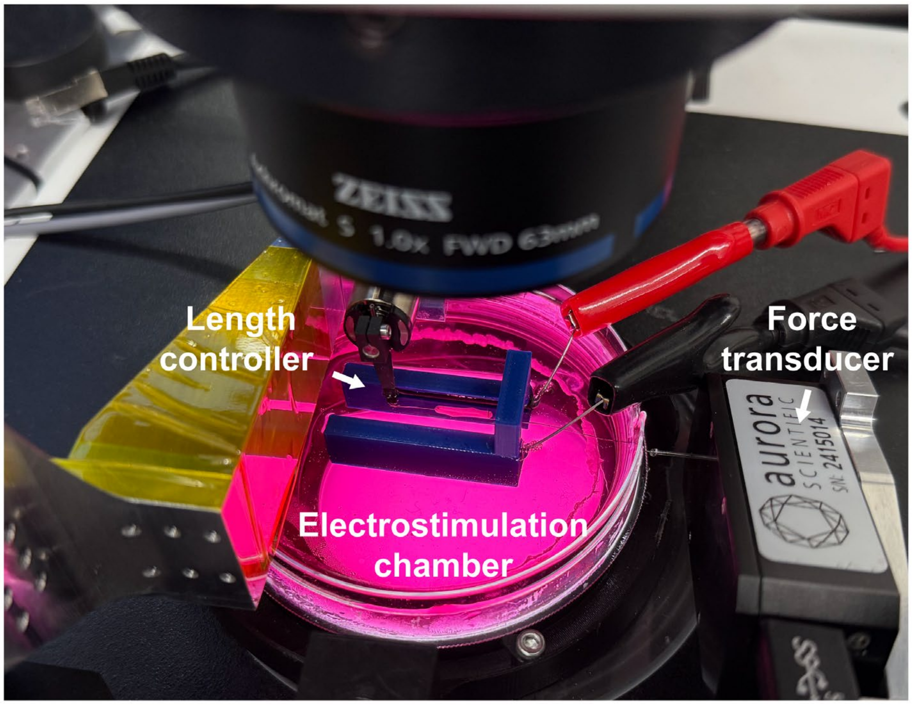

Figure S6. Photograph of the force characterization setup.

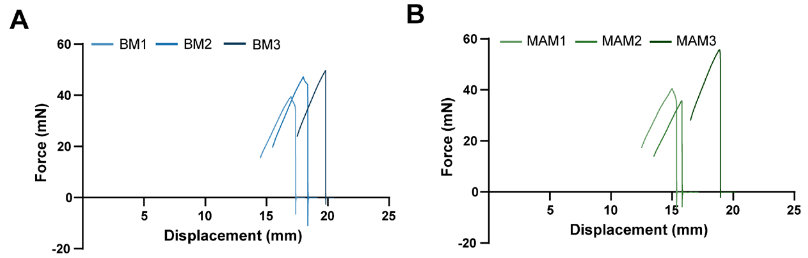

C

	L_0 (mm)	Strain-Rate (s^{-1})	E-Modulus (kPa)
BM 1	13.028	0.0182	47.54
BM 2	13.700	0.0182	55.29
BM 3	14.214	0.0176	59.48
MAM 1	12.000	0.0208	43.15
MAM 2	13.250	0.0189	36.52
MAM 3	14.340	0.0174	67.89

Figure S7. Characterization of Elastic modulus. Force-displacement measurements of BM **(A)** and MAM **(B)** samples depicting elongation before rupture. **(C)** Table summarizing L_0 (calculated by extrapolating the linear section of the measured data to the y-intersection), the calculated strain rate based on the constant displacement rate of 0.25 mm per second and L_0, and the elastic modulus calculated as nominal stress divided by nominal strain, for each of the samples.

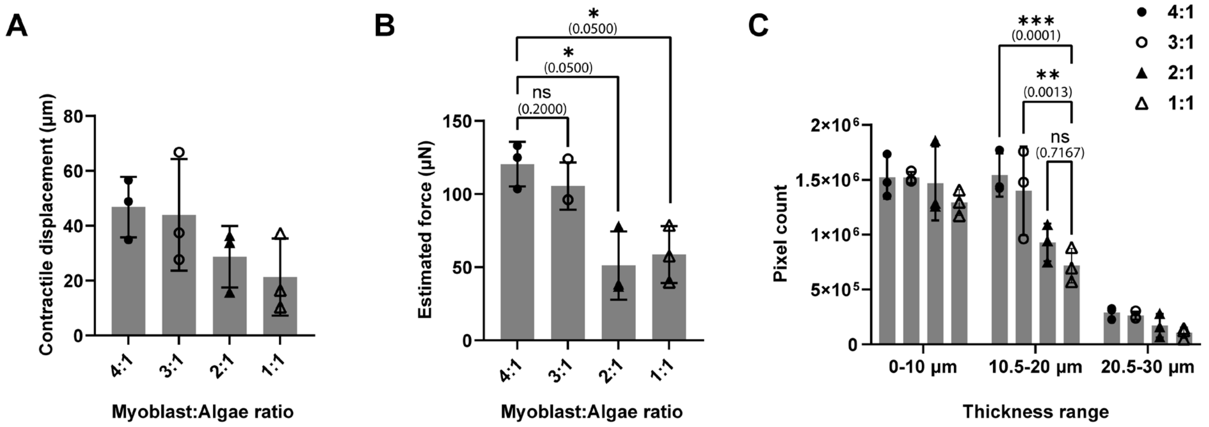

Figure S8. Optimization of myoblast:algae ratio in MAM. (A) Comparison of maximum measured displacement of MAM fabricated with different ratios of myoblast:algae. Data is presented as mean ± SD, N = 3 biological replicates. **(B)** Comparison of active force produced by MAM fabricated with different ratios of myoblast:algae, which was calculated based on the maximum displacement of the scaffolds. Data is presented as mean ± SD, N = 3 biological replicates. Statistical significance was determined using the nonparametric Mann–Whitney U test. Not significant (ns) P > 0.05, *P ≤ 0.05. **(C)** Comparison of myotubes thickness of MAM fabricated with different ratios of myoblast:algae. Immunofluorescence staining using an anti-Myosin antibody was performed to visualize the myotubes. Data is presented as mean ± SD, N = 3 biological replicates. Statistical significance was determined using the two-way ANOVA test. Not significant (ns) P > 0.05, **P ≤ 0.01, ***P < 0.001.

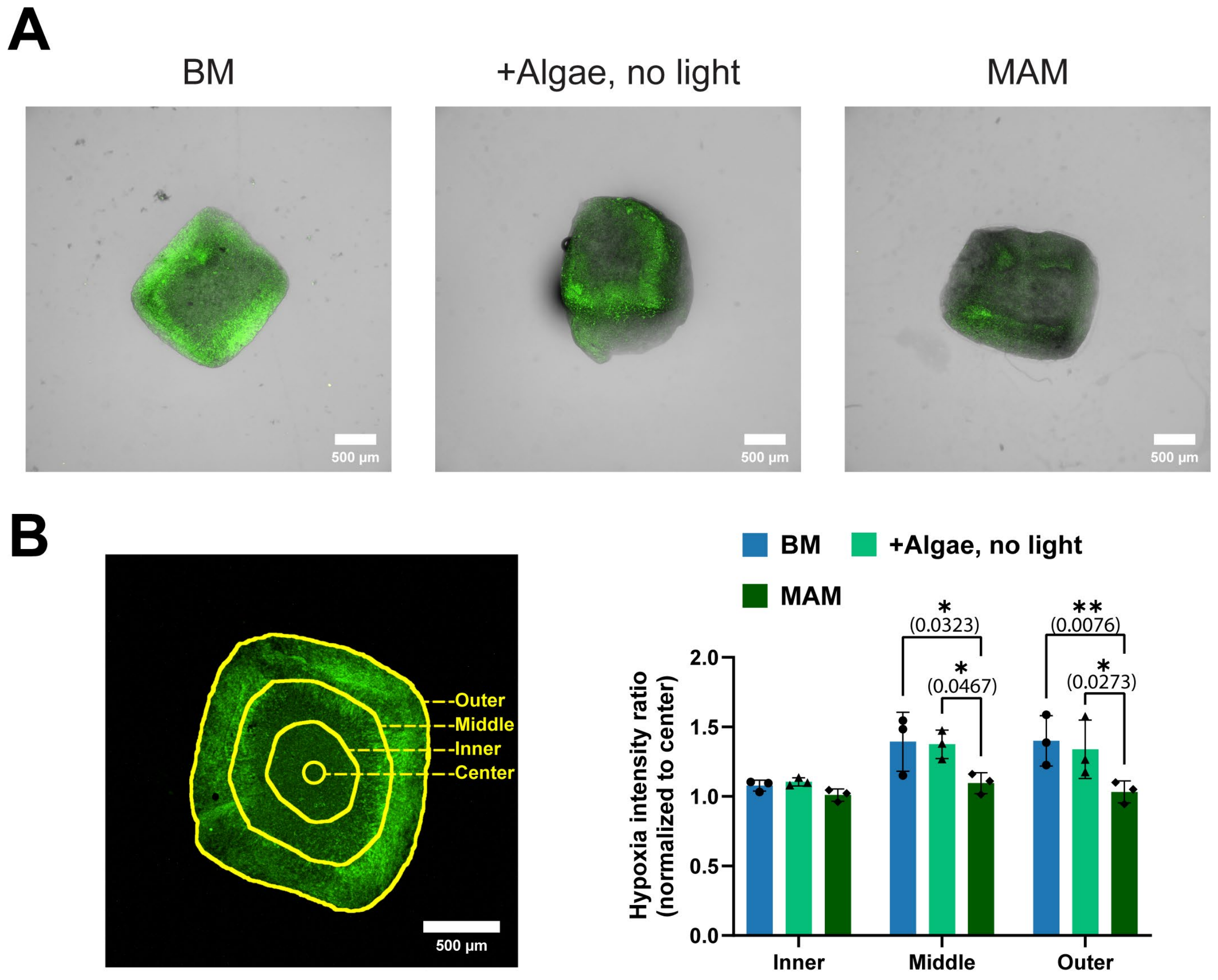

Figure S9. Spatial analysis of hypoxia in muscle constructs. (A) Overlay of bright field and fluorescence microscopy images of muscle construct cross-sections stained with hypoxia reagent. **(B)** Cross-section divided into four regions: center, inner, middle, and outer, for spatial quantification of hypoxia (left). Fluorescence intensity in each region normalized to that of the center to obtain a spatial hypoxia ratio (right). Data is presented as mean ± SD, N = 3 biological replicates. Statistical significance was determined using the two-way ANOVA test, $*P \leq 0.05$, $**P < 0.01$.

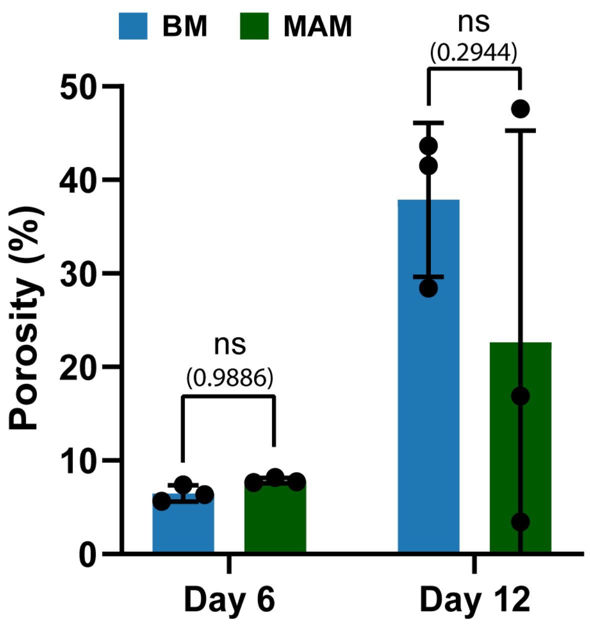

Figure S10. Porosity study of BM and MAM on day 6 and day 12. Results are shown as the percentage (%) of the pore area measured from brightfield images of muscle ring cross sections (10 μm).

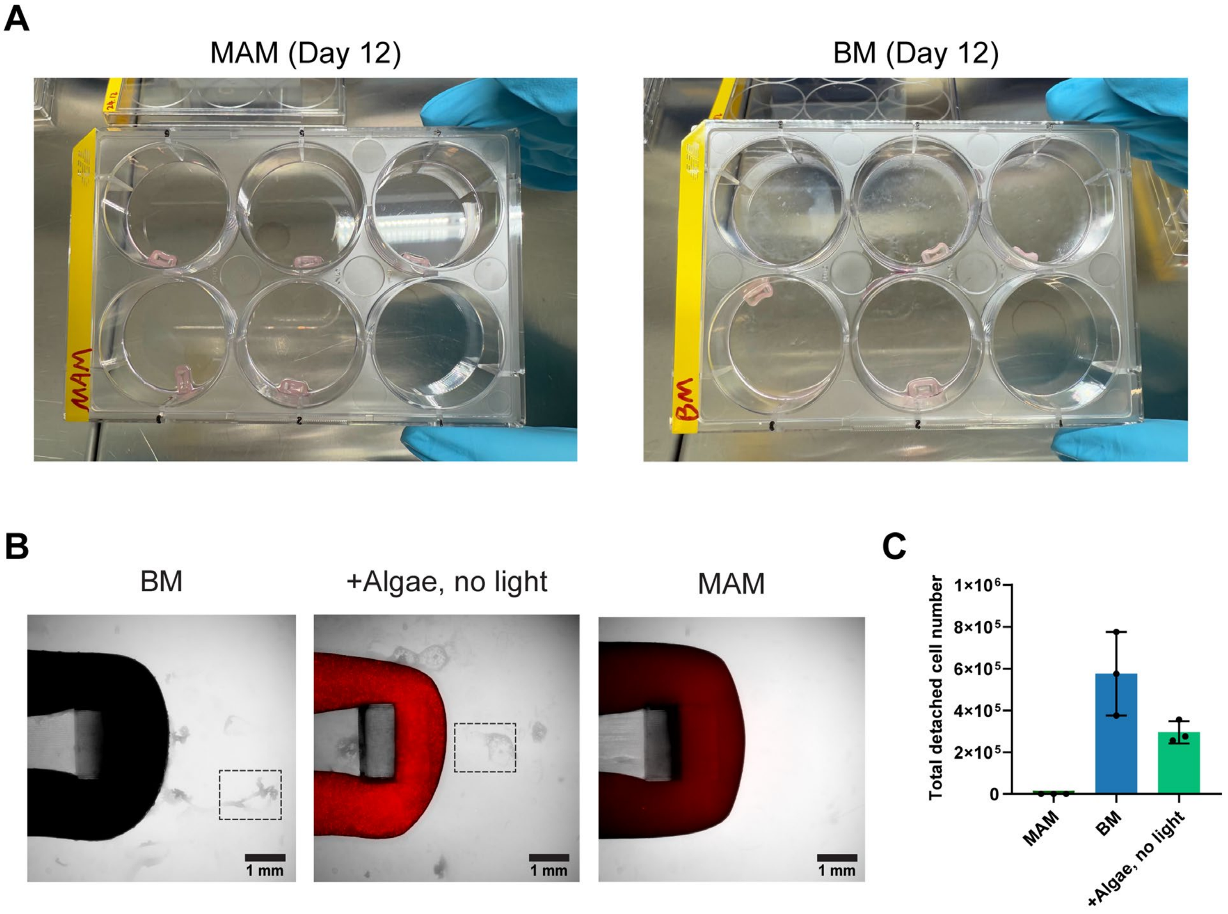

Figure S11. Cell detachment from muscle constructs. (A) After 10 days of differentiation (day 12), the differentiation medium was removed. A detached cell layer was observed in the culture plates containing BM, whereas the culture plates with MAM remained clean, without visible cell layer. **(B)** Optical microscopy images showing the presence of detached cells from BM, microalgae-embedded constructs without light (+Algae, no light), and MAM. The dashed squares represent examples of adherent cells detached from the muscle constructs. **(C)** Quantification of the total number of detached cells presented in the culture plates (bottom right).

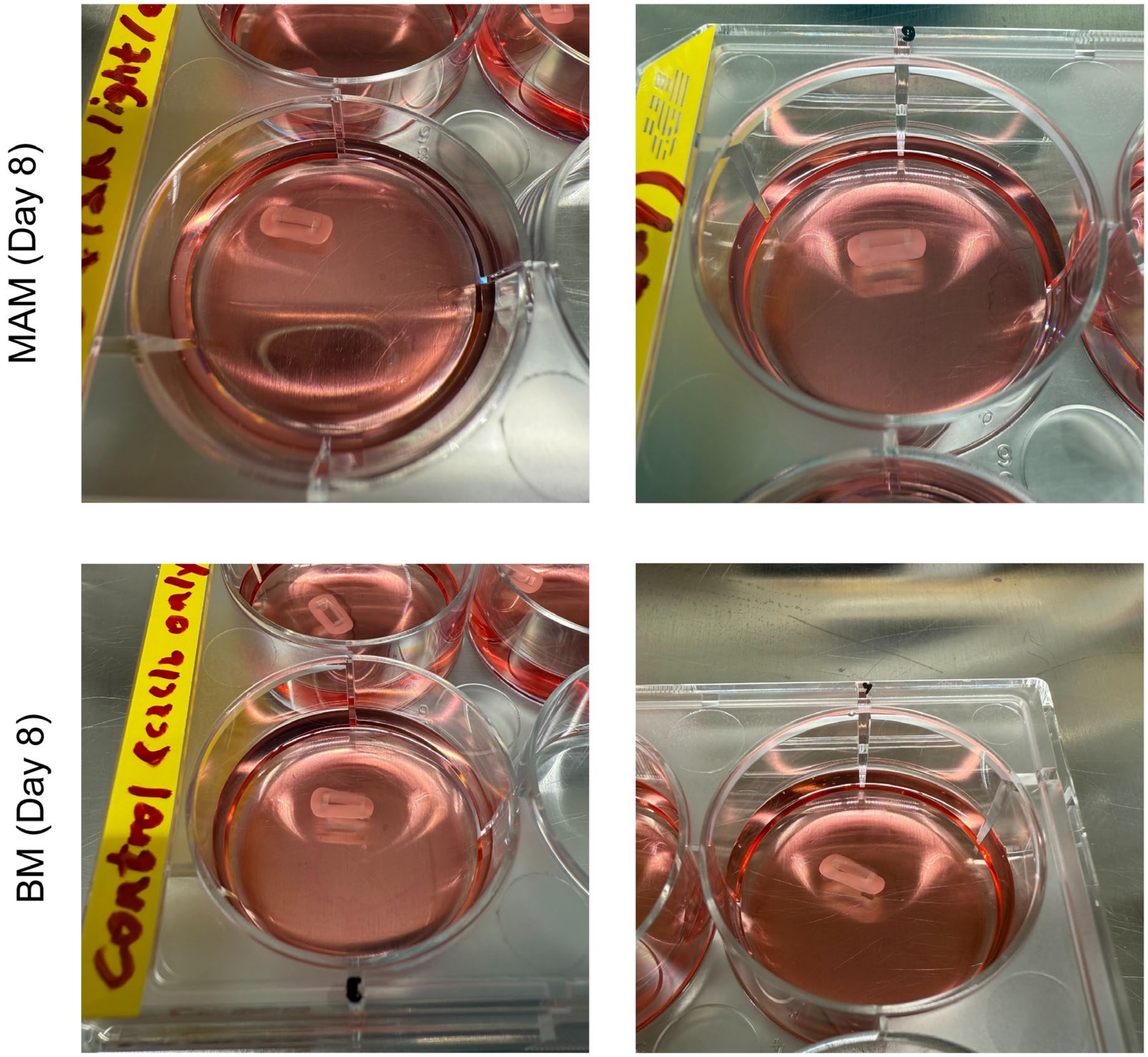

Figure S12. Photographs of MAM and BM on day 8. After 6 days of differentiation, MAM maintained a uniform shape, whereas BM displayed visible twisting.

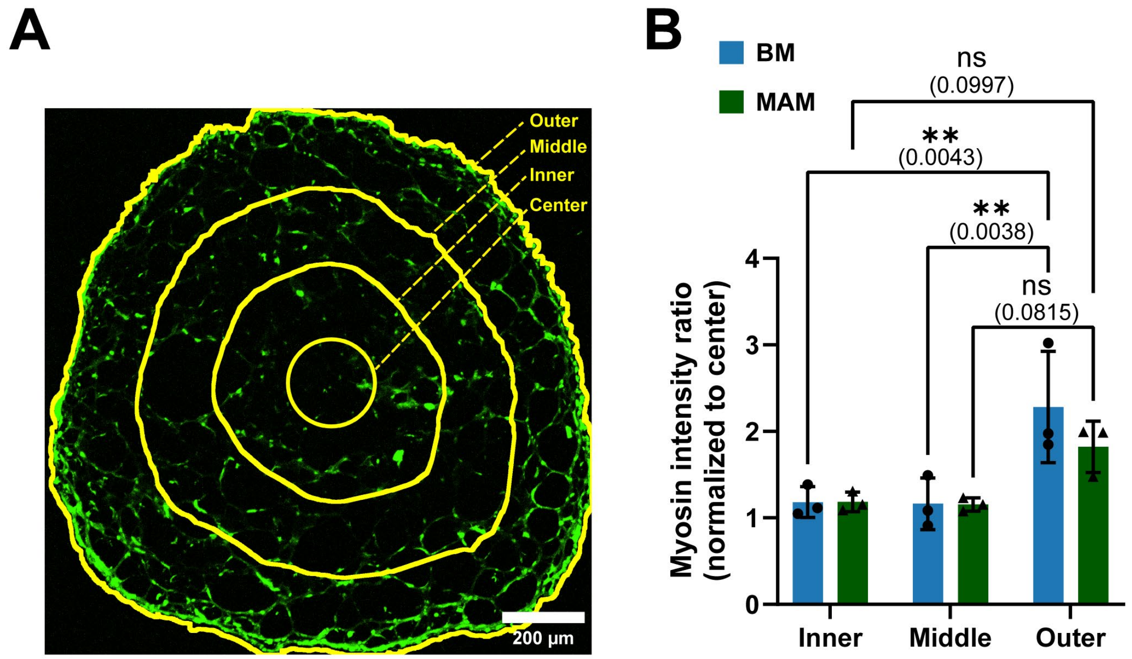

Figure S13. Spatial analysis of myosin in BM and MAM. (A) Cross sections divided into four regions: center, inner, middle, and outer, for spatial quantification of myosin. **(B)** Fluorescence intensity in each region normalized to that of the center to obtain a spatial myosin intensity ratio. Data is presented as mean ± SD, N = 3 biological replicates. Statistical significance was determined using the two-way ANOVA test. Not significant (ns) $P > 0.05$, $**P < 0.01$.

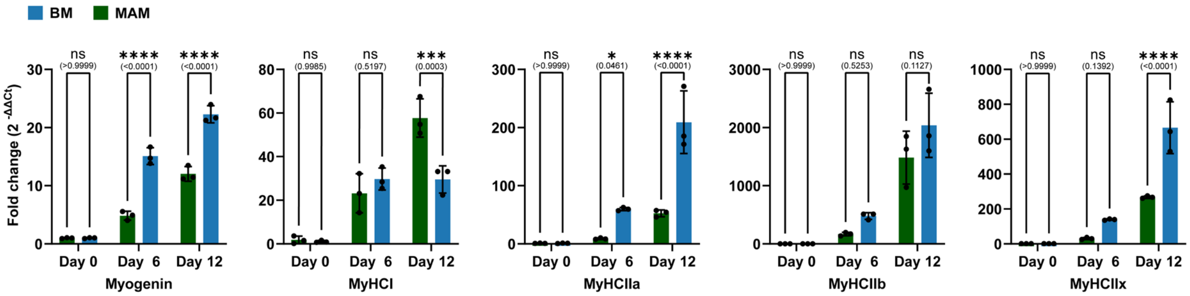

Figure S14. RT-qPCR analysis of MAM and BM normalized against the RPS12 housekeeping gene. (**A**) Analysis of myogenic biomarkers on day 0, 6 and 12. Data is presented as mean ± SD, N = 3 biological replicates. Statistical significance was determined using two-way ANOVA. Not significant (ns) P > 0.05, *P ≤ 0.05, ***P < 0.001, ****P < 0.0001.

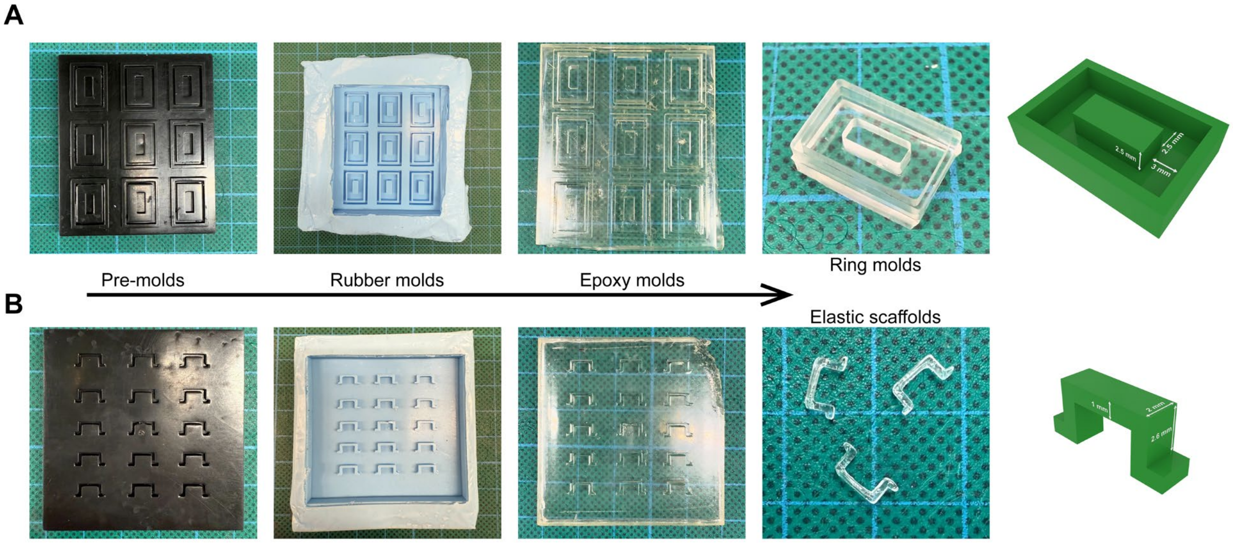

Figure S15. Photographs showing the fabrication process of the ring mold (A) and elastic scaffold (B).

Genes	Nucleotide sequences (5'-3')	References
GAPDH	FW: ATGGTGAAGGTCGGTGTGAA	*21*
	RV: GAGGTCAATGAAGGGGTCGT	
RPS12	FW: AAGGCATAGCTGCTGGAGGTGTAA	*47*
	RV: AGTTGGATGCGAGCACACACAGAT	
Myogenin	FW: CCCTACAGACGCCCACAATC	*21*
	RV: ACCCAGCCTGACAGACAATC	
MyHCI	FW: GCCCCAAGCACAAGGAGT	*21*
	RV: AGCCCCAAGAAATAAGGACAG	
MyCIIa	FW: GCAGAGACCGAGAAGGAG	*21*
	RV: CTTTCAAGAGGGACACCATC	
MyHCIIb	FW: GAAGGAGGGCATTGATTGG	*21*
	RV: TGAAGGAGGTGTCTGTCG	
MyHCIIx	FW: GCGACAGACACCTCCTTCAAG	*21*
	RV: TCCAGCCAGCCAGCGATG	

Table S1. Nucleotide sequences of RT-qPCR primers and corresponding references.

Movie S1. Muscle contraction of MAM and BM induced by electrical stimulation at a frequency of 1 Hz.
